# Minimally invasive transoral surgical approach to the medial compartment of the masticator space for venous malformation and benign tumor removal

**DOI:** 10.3389/fsurg.2026.1691677

**Published:** 2026-04-07

**Authors:** Riccardo Nocini

**Affiliations:** Head and Neck Department, Azienda Ospedaliera Universitaria Integrata di Verona, Verona, Italy

**Keywords:** coronoid process, mandibular notch, masticator space, n-butyl-2-cyanoacrylate, venous malformation

## Abstract

**Background:**

This article describes a new surgical technique to approach a venous malformation and benign tumors located in the medial compartment of the masticator space.

**Methods:**

A 50-year-old woman reported painless swelling of the buccal mucosa above the medial surface of the right ramus. A CT scan and MRI imaging suggested a neuroma-like lesion. A radiological examination could not determine the nature of the lesion, and therefore, a convincing diagnosis could not be achieved. Considering the focal bone erosion at the mandibular notch, the uncertain diagnosis, and especially the impossibility of excluding other benign lesions of the masticator space, the patient underwent a surgical removal of the lesion.

**Results:**

The hypothesized neuroma turned out to be a venous malformation that was confirmed clinically and histologically. When the diagnosis is uncertain, only a histological examination can determine the true nature of the lesion. The presently described technique considered mouth opening issues: when the mandibular ramus rotates and moves forward, the coronoid process is anatomically close to the tuber maxillae. Therefore, there is not enough space to approach the medial compartment of the masticator space unless the coronoid process is temporarily removed. An L-shaped osteotomy of the coronoid process is minimally invasive, safe, and comfortable. Coronoidotomy offers a wide field of vision and allows easy transoral access to the masticator space.

**Conclusions:**

Combined mandibular coronoidotomy, with or without endoscopic magnification, is a suitable approach to treat a well-defined lesion in the medial compartment of the masticator space with the involvement of the mandibular ramus; intralesional injection of n-butyl-2-cyanoacrylate in a venous malformation completes the surgical procedure in a predictable and safe way.

## Introduction

The masticator space is a complex anatomical region composed of the mandibular ramus and the masticatory muscles: masseter, medial, and lateral pterygoid muscles and the caudal region of the temporalis muscle. The mandibular ramus divides the space into medial and lateral compartments (1, 2). Anteriorly, the masticator space borders on the buccal space, posterolaterally, it borders on the parotid space, medially, it confines itself within and corresponds to the apical region of the parapharyngeal space, and finally, inferomedially, it borders on the submandibular space. The masticator space connects with the middle cranial fossa via the oval foramen and with the pterygopalatine fossa (PPF) via the pterygomaxillary foramen, and superiorly, it continues in the infratemporal fossa that represents its superior theoretical border (1, 3).

Considering its anatomic features, a physical examination of the masticator space may pose tremendous challenges (3). Lesions originating in this deep-lying space are commonly diagnosed late because of their non-specific symptoms. Moreover, masses growing in the infratemporal fossa, and therefore in the masticatory space, frequently correspond to the extension of lesions originating from adjacent anatomic compartments. These lesions may originate from the paranasal sinuses, the middle cranial fossa, the parotid gland, and the pharynx. While primary tumors are rare and usually benign (1), metastases in this area are rarely encountered (2). Schwannoma or neurinoma is the most frequently occurring benign tumor of the masticator space, and vascular anomalies are the second most frequently encountered neoplasm (1).

In this technical note, the author aims to present the correct diagnostic pathway and surgical approach of a benign lesion located in the medial compartment of the masticator space. A clinical case of a patient suffering from a tumor involving the mandibular ramus, with radiological features suggestive of schwannoma, is presented. This type of mandibular coronoidotomy, with or without endoscopic magnification, is a suitable approach to completely eradicate a well-defined lesion of the masticator space. To the best of our knowledge, this surgical approach has not been described in the literature yet.

## Case report

A 50-year-old Caucasian woman with negative medical history was referred to the ENT outpatient section of the Unit of Otolaryngology of our University Hospital. The patient reported painless swelling in the masticator space associated with a sense of pressure in the area under the zygomatic arch. Upon a preliminary clinical evaluation, the patient's face showed no discernible alterations: there were no skin lesions on the cutaneous lining, and face symmetry was maintained. No changes under the functional point of view were assessed, either for the seventh cranial nerve or for the fifth cranial nerve pairs, respectively. Bone profiles were maintained, and the mucosal lining of the oral cavity appeared within the range of normality. Occlusion was preserved, and the mandible kinetics was unaltered.

**Figure 1:**
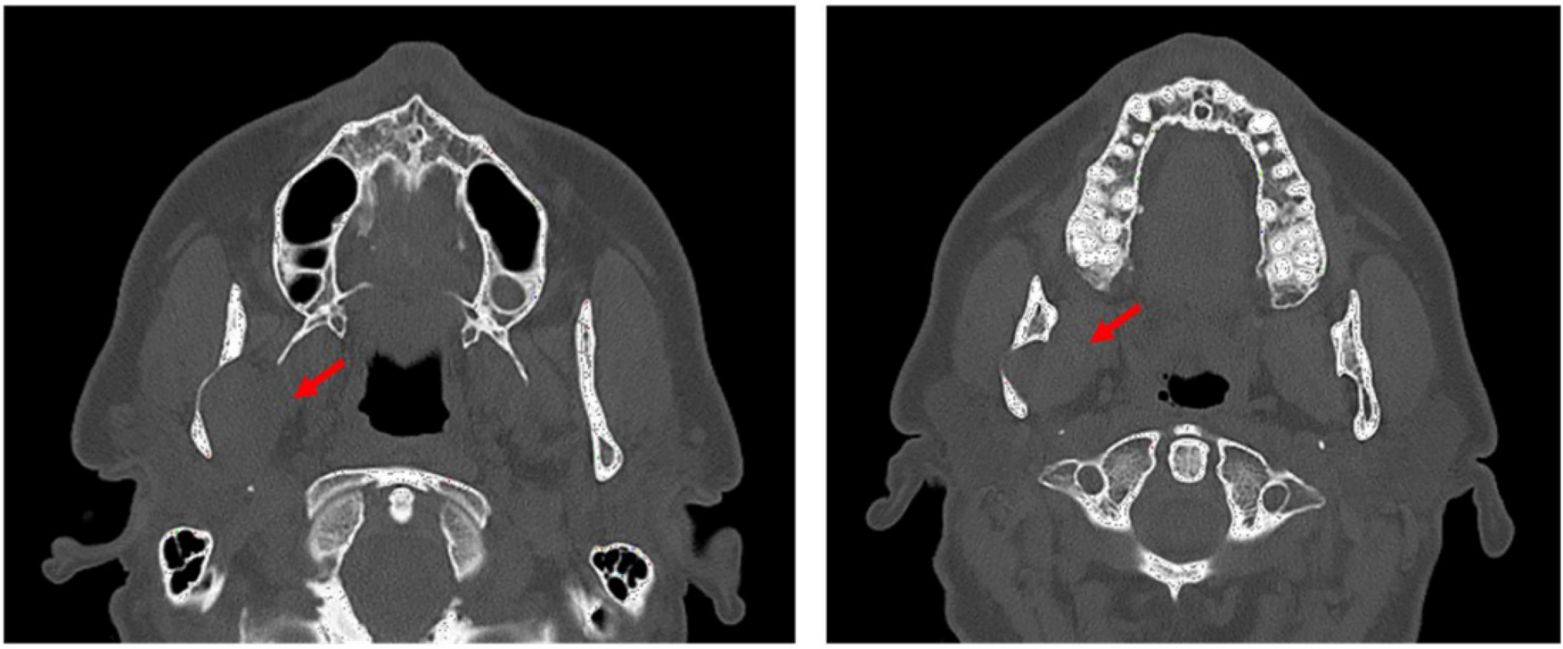
Preoperative CT reveals an ovoid well-defined lesion measuring 2.5 cm located in the right medial compartment of the masticator space, with scalloping over the inner surface of the mandibular ramus, near the mandibular notch and over the Spix foramen. There are no signs of invasion of adjacent anatomical structures.

To complete clinical evaluation and to precisely determine the type of lesion and its anatomical localization, the ENT team was required to perform a computed tomography (CT) scan and magnetic resonance imaging (MRI): a more detailed study of the region and a definition of the anomalous swelling origin were mandatory. The CT scan (Figure 1) documented a lesion of the right masticator space with erosion of the inner surface of the mandibular ramus, at the level of the mandibular notch.

**Figure 2:**
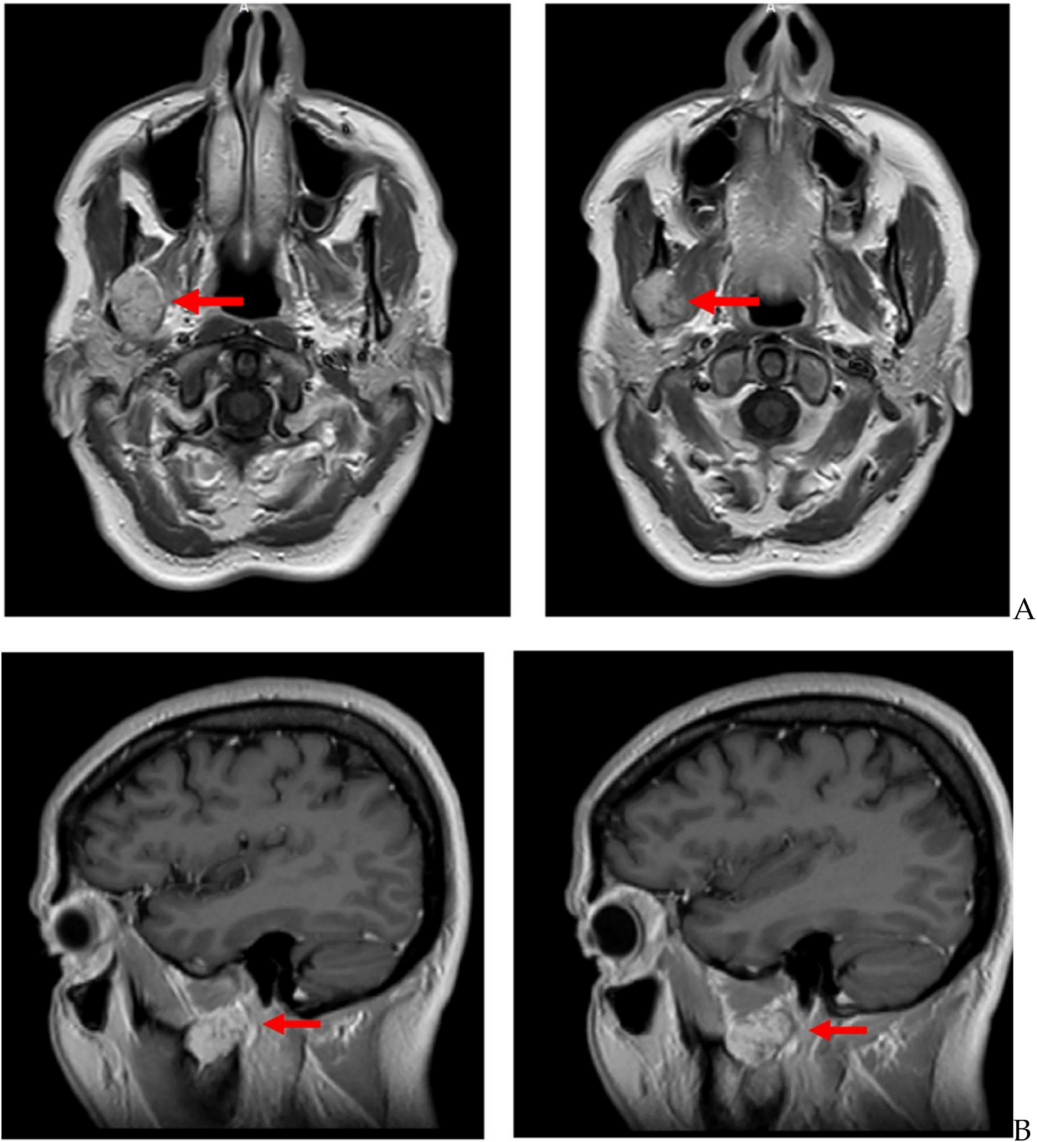
**(A)** preoperative MRI with a contrast medium; axial T1 fat sat sequence reveals an ovoid, lobed, encapsulated, and well-defined lesion measuring 2.5 cm in the right medial compartment of the masticator space, with heterogeneous T1 signal and late heterogeneous enhancement. There are no signs of invasion of the adjacent structures of anatomical spaces. **(B)** Preoperative MRI with a contrast medium; sagittal T1 fat sat sequence reveals an ovoid, lobed, encapsulated, and well-defined lesion measuring 2.5 cm in the right medial compartment of the masticator space, with a heterogeneous T1 signal and late heterogeneous enhancement. There are no signs of invasion of adjacent structures or anatomical spaces.

The MRI (Figures 2A,B) identified a 2.5-cm solid lesion with non-homogeneous postcontrast enhancement, located medially to the right mandibular branch, between the medial and the lateral pterygoid muscles.

**Figure 3:**
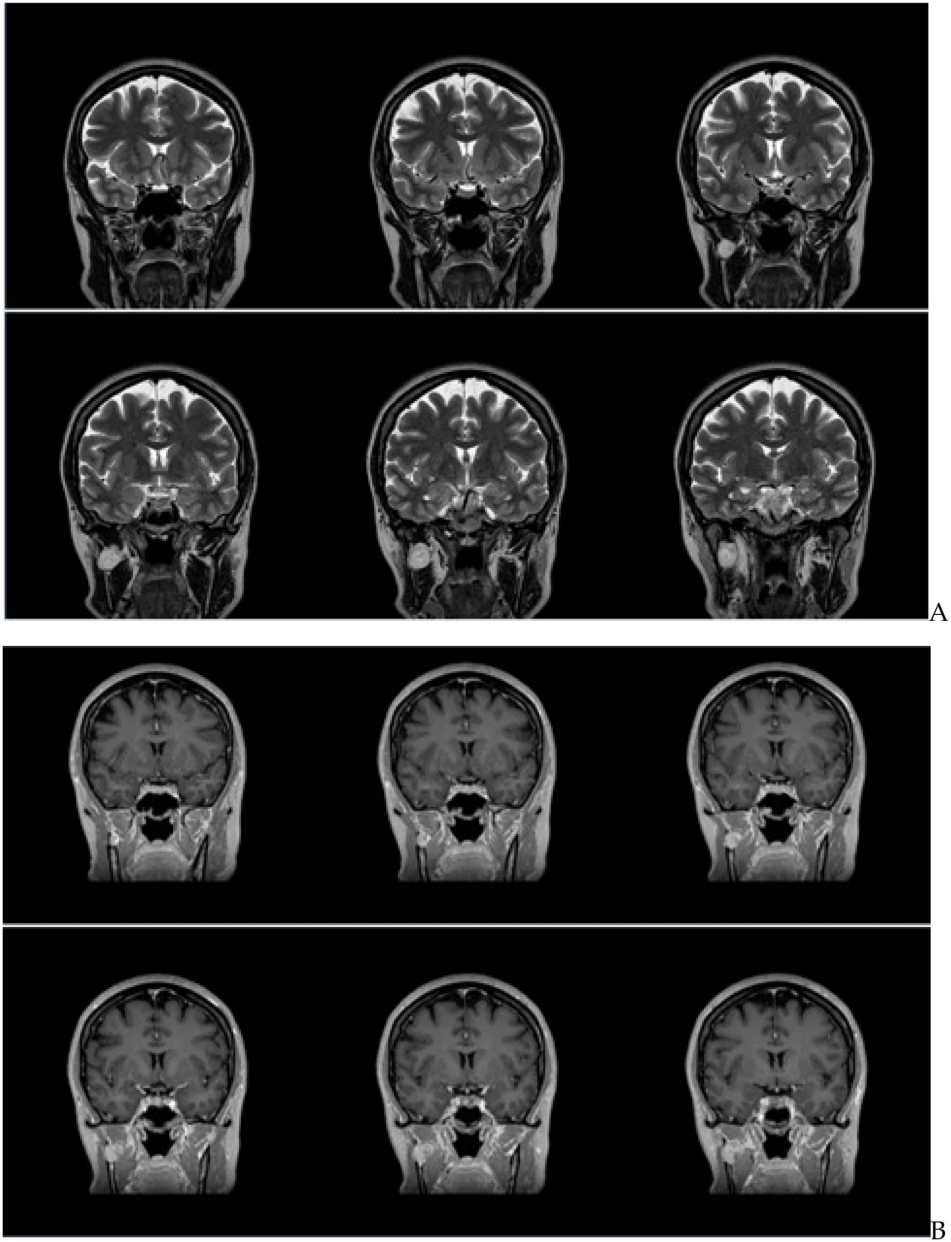
**(A)** MR T2-weighted. **(B)** MR T1-weighted.

The MRI showed that there were no infiltrative patterns toward the adjacent muscular structures, while bone remodeling and thinning of the mandibular branch could be identified. The radiologist concluded that the lesion was suggestive of a schwannoma of the masticator space (Figures 3A,B).

An ultrasound examination was not performed, as the tumor position was difficult to achieve both from the cutaneous side and from the intraoral position.

Considering the focal bone erosion at the level of the mandibular notch, the lack of diagnostic certainty radiologically assessed, the benign radiological features, and the simultaneous impossibility of excluding malignant lesions of the masticator space without a histological definition, the surgical team decided to perform a surgical excision of the tumor and a histological examination.

## Surgical technique

The patient received general anesthesia with nasotracheal intubation. First, the mucosal and submucosal tissues of the first quadrant were infiltrated with local anesthetic and a vasoconstrictor. To reach the medial compartment of the masticator space, a mucosal incision following the projection of the external line of the right mandibular ramus was performed. The incision was completed with a mesial vestibular release at the level of 4.7 and distal discharge to the right retromolar triangle. A mucoperiosteal flap was elevated from the angle of the mandible laterally, and from the ascending mandibular ramus medially, to adequately expose both the ramus and the coronoid process. To provide a better visibility of the lingula, the attachment of the temporalis muscle was transected, and the coronoid process was osteotomized using a Mectron piezoelectric scalpel, an instrument that has largely proven to be respectful of soft tissue and the periosteum in narrow anatomical sites (4–6). An L-shaped osteotomy line was drawn with a pencil on the lateral face of the mandibular ramus, as shown in Supplementary Figure 1A. The vertical branch of the “L” was executed from the mandibular notch up to the inferior third of the mandibular ramus, at the level of the amelocemental junction of the last inferior molar. The horizontal branch of the “L” was executed from the caudal extremity of the vertical branch to the anterior edge of the temporal crest, running exactly at the base of the coronoid process.

**Figure 4:**
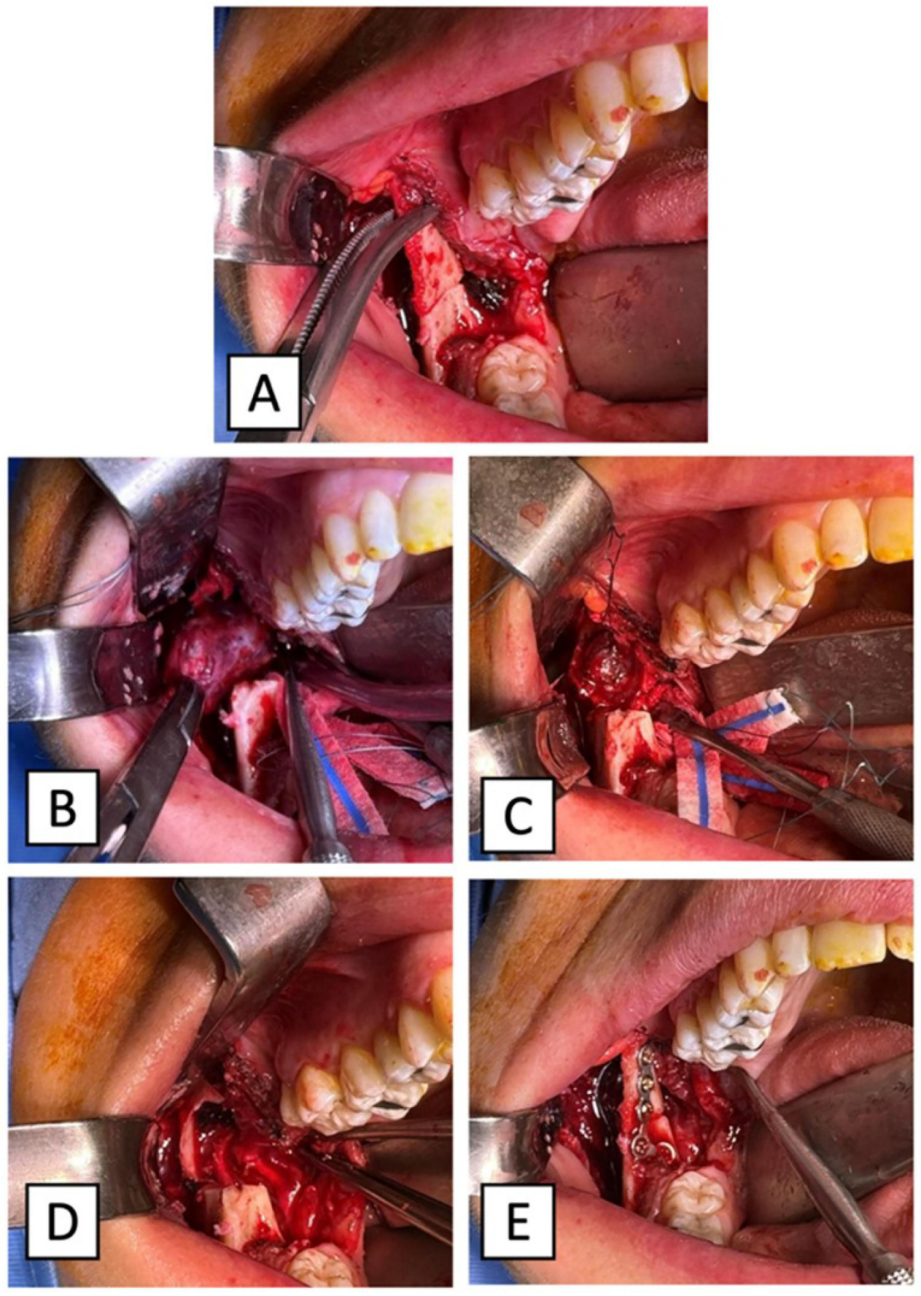
**(A)** Intraoperative view of the lesion after mandibular coronoid process osteotomy. **(B)** Intraoperative view of the lesion after the intralesional injection of n-butyl-2-cyanoacrylate proves that the venous malformation solidifies and it is possible to remove it entirely without bleeding. **(C)** The remaining scalloping over the mandibular ramus is now clearly detectable. **(D)** The coronoid process is repositioned. **(E)** A titanium microplate is placed to promote the healing of the coronoid process.

Preplating of the right coronoid process was executed using a five-hole L-shaped titanium microplate with its screws, paying attention to distributing at least two holes per each side of the osteotomy to ensure fixation stability. Figure 4 illustrates the surgical technique step by step. Once the coronoidotomy was completed (Supplementary Figures 1B–D and Figures 4A–E), the new growth was completely exposed and it was visually identified as a venous malformation (VM) (Figure 4B).

**Figure 5:**
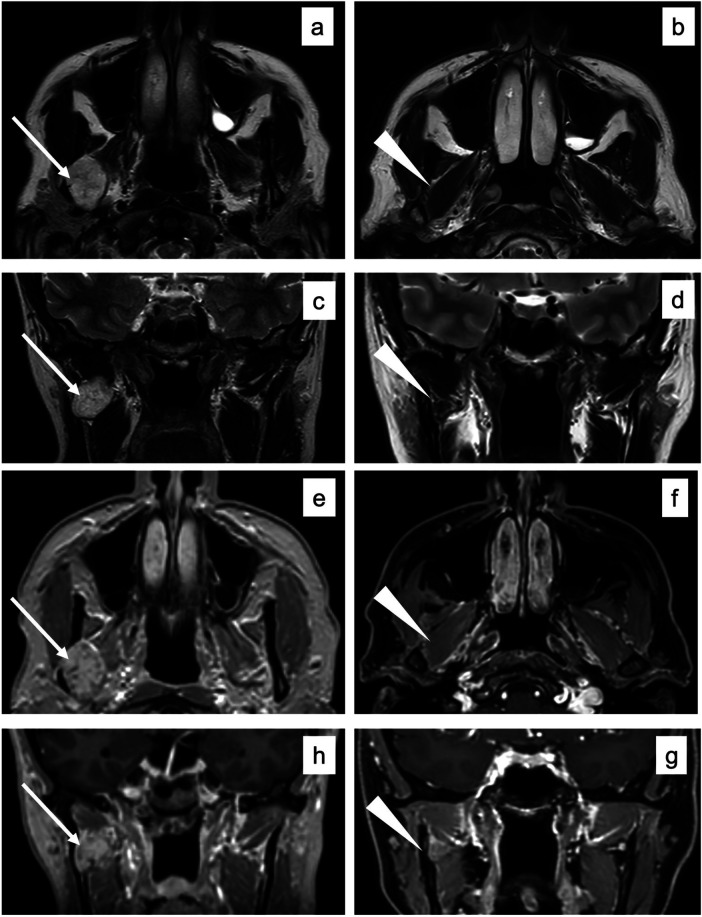
Axial **(a,b)** and coronal **(c,d)** magnetic resonance T2-weighted imaging without fat suppression and T1-weighted axial **(e,g)**, without fat suppression, and coronal **(f,h)**, with fat suppression. In presurgical images, a hyperintense round margin hypervascular mass is evident in the masticator space, located posteriorly to the distal part of the temporal muscle, medially to the mandibular ramus, and laterally to the lateral pterygoid muscle **(a,c,e,g)** arrows). In postsurgical images, the mass is completely removed, with restitutio ad integrum of the masticator space and its structures (**b,d,f,g**, arrowheads).

The VM was rated following two grading systems for venous malformations. It was classified as Type I according to Puig et al. (4, 5) and rated as “common venous malformation” according to the ISSVA system (6). The VM was in close relationship with the right inferior alveolar nerve; however, the two structures were easily dissociable. To avoid intraoperative bleeding of the venous malformation and facilitate dissection from adjacent structures, the mass was directly injected and embolized with n-butyl-2-cyanoacrylate (Glubran). Once the lesion solidified, it was possible to remove it entirely, without bleeding and preserving all the noble structures. The osteotomized coronoid process was fixed rigidly in its prior anatomical position with the five-hole microplate and relative 5 mm screws that were preplated. The five-hole titanium microplate was fixed in the easiest possible position: on the medial face of the coronoid process and on the right retromolar fossa. Soft tissue closure was performed. In Figures 5a–h, presurgical and postsurgical MR images describe the procedure. In presurgical images, a hyperintense round margin hypervascular mass is evident in the masticator space, located posteriorly to the distal part of the temporal muscle, medially to the mandibular ramus, and laterally to the lateral pterygoid muscle (Figures 5a,c,e,g, arrows). In postsurgical images, the mass is completely removed, with full restoration of the masticator space and its structures (Figures 5b,d,f,h, arrowheads).

**Figure 6:**
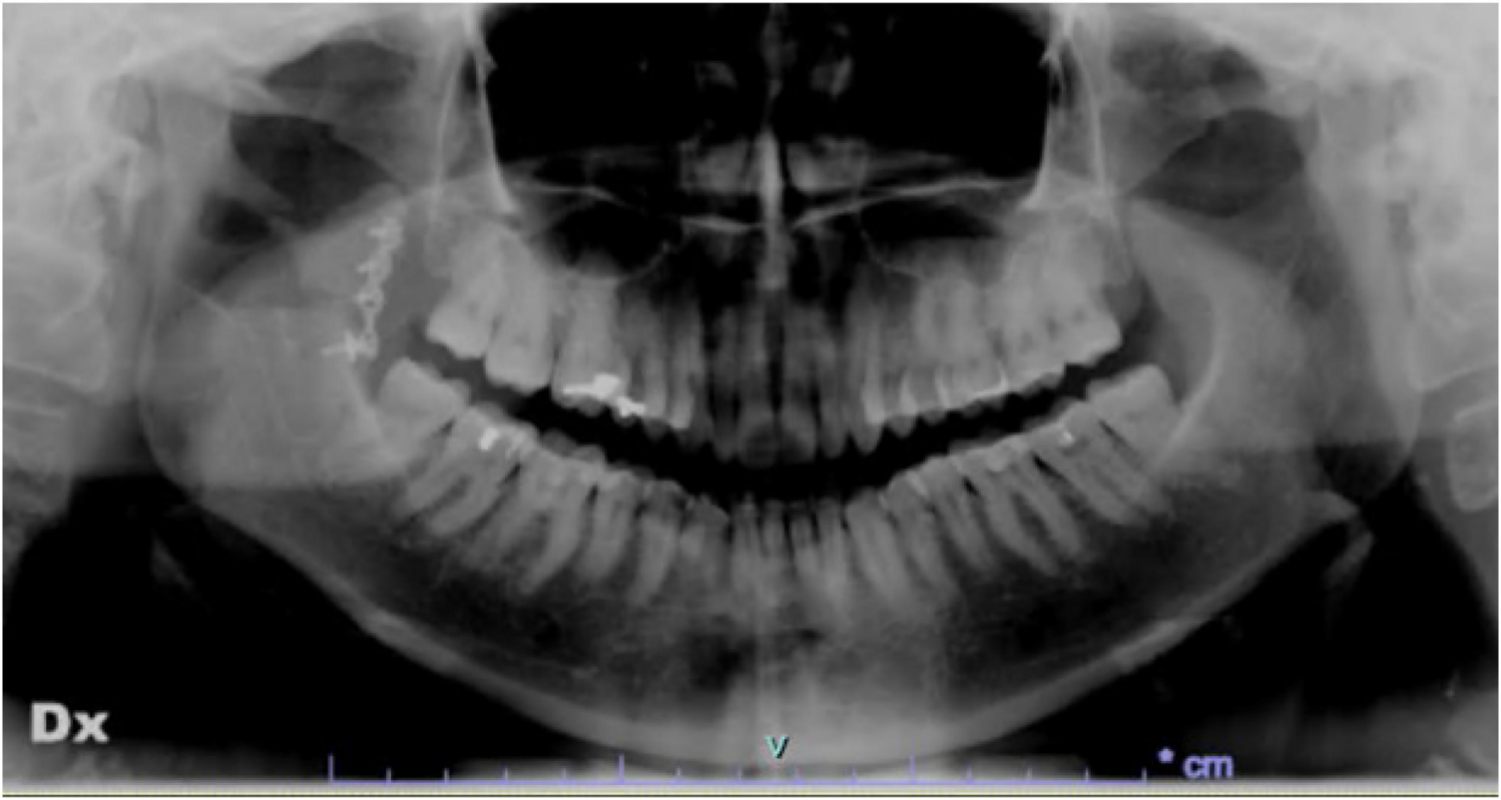
Postoperative orthopantomography (1 week after surgery).

**Figure 7:**
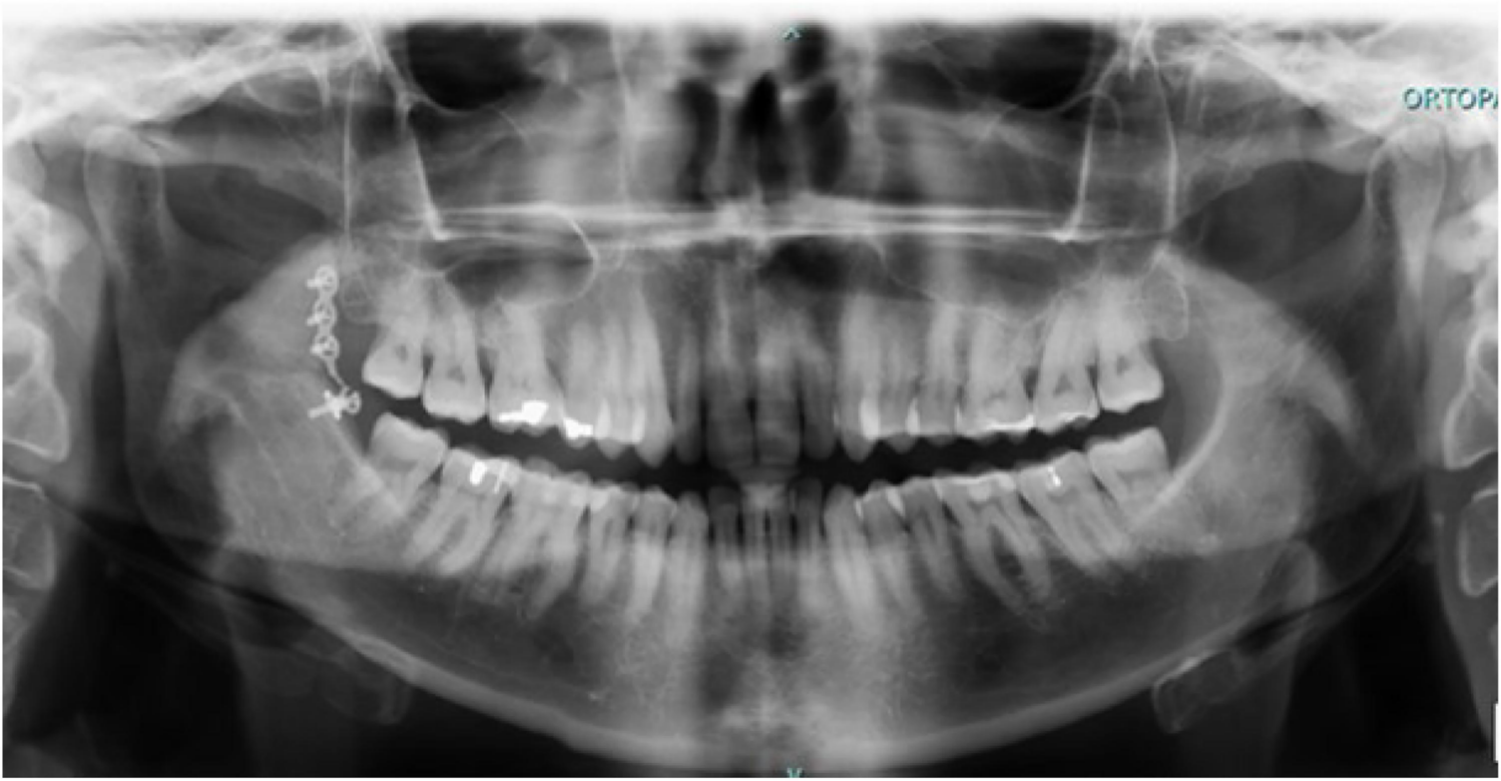
Postoperative orthopantomography (6 months after surgery).

The patient underwent postoperative X-ray imaging (orthopantomography) to verify the correct repositioning of the coronoid process (Figures 6,7).

Reports on the management of coronoid fractures are not univocal in the literature (7–10): our preference is to restore the mandibular anatomy to avoid mouth-opening restrictions, possible adhesions to the surrounding tissues, or jaw deviation as surgical sequelae. After a period of modest paresthesia of the inferior alveolar nerve, the patient completely recovered. This demonstrated that the surgical approach was adequate and reproducible and that the vascular malformation was gently detached from the noble structures of the masticator space, avoiding major damage.

## Discussion

The pathology of the masticator space varies from inflammation to benign tumors, from vascular anomalies to malignant cancer. Primary tumors develop from the nerves, the bone, or the soft tissues that effectively constitute the masticator space. Alternatively, secondary tumors extending toward the masticator space may be diagnosed; in these patients, tumors originate from adjacent anatomical sites and extend to the masticatory space through direct or indirect paths.

Within the group of masticator space benign tumors, schwannomas are the most frequently occurring tumors. Schwannomas are slow-growing, encapsulated tumors developing from Schwann cells (11, 12). Radiologically, schwannomas are isointense or hypointense on T1-weighted imaging and hyperintense on T2-weighted imaging. They appear significantly and homogeneously enhanced in the postcontrast phase. These features were strongly evident in the clinical case presented above. Another benign entity is lipomas, which appear as uniform, fatty, low-attenuated lesions on CT scans. On MR T1-weighted images, they exhibit a consistent high signal intensity that diminishes on fat-suppressed images. No contrast enhancement is observed in lipomas.

Tumors extending toward the masticator space may achieve this area via fascia (oropharyngeal tumors) or bone (maxillary lesion with disruption of the posterior maxillary wall) or via vascular o neural foramina (tumor originating from the middle or the anterior cranial fossa). Tumors may also extend to the masticator space from the deep pole of the parotid gland or from the cheek skin, passing through the masseter muscle. Pharyngeal lesions and maxillary carcinomas are the most represented tumors with secondary extension to the masticator space, while nasopharyngeal angiofibroma [a rare and highly vascularized tumor, found almost exclusively in young men (13)] arises from the sphenopalatine foramen and has the tendency to expand to the nasopharynx and nose, as well as to the infratemporal fossa and masticator space. Perineural growth is another possible path to the masticator space, especially for the adenoid cystic carcinoma (1, 2). Another locally aggressive tumor is ameloblastoma, which can extend into the masticator space (8).

Among the primitive malignant tumors of the masticatory space, we find sarcomas (e.g., osteosarcoma, rhabdomyosarcoma, malignant fibrous histiocytoma, fibrosarcoma, and angiosarcoma) and lymphomas, which are usually diagnosed in immunodeficient population; metastasis to the area from tumors localized anywhere else is rare.

Finally, masticator space pathology must always take into account vascular anomalies. Vascular anomalies include tumors (that may regress with the patient’s age) and vascular malformations (which are present at birth, tend to increase in size, and never regress on their own) (6, 7, 14). Their treatment is usually customized upon patient needs (15), and they are classified into benign—locally aggressive, borderline, or malignant tumors (6). To elaborate, vascular anomalies may be capillary, venous, lymphatic, arteriovenous, or combined malformations, depending on the dominant vessel type involved (16, 17). VMs are the most common vascular anomalies (70%) (7). When vascular anomalies are localized in deep tissues or are very small-sized, they may remain undetected for years or decades (7).

To correctly classify a vascular anomaly, it is fundamental to consider the patient's clinical history and, above all, it is fundamental to perform appropriate imaging (18). On ultrasound, venous malformations are characterized by a slow or absent flow pattern. MRI plays an important role in the diagnosis and follow-up of MVs: they are usually hyperintense on T2-weighted images and isointense with muscle tissue in precontrast T1-weighted images. Diversely, they show hyperintensity in postcontrast T1-weighted images, which is naturally due to the contrast agent pooling inside the lesion. Calcific phleboliths in the venous malformation area may appear as a sign of previous intravascular thrombosis (6, 16).

A surgical approach to lesions located in the masticator space may represent a challenge to the surgeon. Early diagnosis plays a major role in lesion management. It is mandatory to carefully consider the risk of morbidity after the procedure and the risk of intraoperative complications. In the literature, different surgical techniques have been described to approach this anatomical area, for example the subtemporal-preauricular approach, the Weber–Ferguson approach, and the transoral approach associated or not associated with mandibulotomy. Mandibulotomy is often required when tumors of the tonsillar region extend into the infratemporal fossa or when nasopharyngeal masses spread into the tonsillar region. The transoral approach has multiple advantages: it provides good access to the surgical site and allows the surgeon to precisely perform the excision of tumors extending posteriorly to the pterygopalatine fossa and infratemporal fossa (2, 11). Attia et al. proposed an approach to the pterygomaxillary fossa and the parapharyngeal space consisting of a transcervical incision combined with double osteotomies of the mandible. This approach allows the ascending ramus to be swung laterally, keeping the neurovascular bundle intact. The drawbacks of this technique are linked to the sacrifice of the external carotid artery, the risk of trigeminal nerve mandibular branch injury, and insufficient exposure of the infratemporal and pterygopalatine fossa (19).

Sekhar described a combined subtemporal and preauricular approach to the infratemporal fossa to reach the middle and posterior skull base. This approach was found to be useful in tumors with intracranial progression. Fisch et al. described a systematic surgical approach to the lesions of the infratemporal fossa and a skull base–like type C approach and type D modification (20). The disadvantages of the Fisch technique are the sacrifice of the Eustachian tubes and injury to the facial nerve, the mandibular and maxillary branches of the trigeminal nerve, and the internal carotid artery. Furthermore, it has frequently been associated with postoperative TMJ dysfunction (6, 16).

Shah et al. reported a surgical approach that includes a hemicoronal incision, continues along the tarsal plates of the upper and lower eyelid, reaches the medial canthus, and ends with a Weber–Ferguson incision dividing the upper lip on the midline. Shah et al.’s approach is indicated for lesions involving the pterygopalatine, infratemporal, and anterior and middle cranial fossa; nevertheless, this is a highly invasive approach (13, 21). The endoscopically assisted transoral approach is described for excision of tumors in the parapharyngeal space; the pathology was often found to be pleomorphic adenoma and schwannoma (22, 23). In the patient case reported in this study, the lesion was located near the Spix spine, without eroding the cortical bone, but with a scalloping over the line surface of the mandibular ramus. Given the anatomical location and the radiological features of the mass, a minimally invasive surgical approach was planned. A transoral approach with mucosal incision extended into the inferior vestibular sulcus posteriorly and then prolonged in the upward direction lateral from the retromolar triangle and the maxillary tuberosity. The rationale of this technique is explained as follows: In mouth opening, the coronoid process finds itself too close to the tuber maxillae, and the intraoral space becomes too narrow to approach the medial compartment of the masticator space. The L-shaped coronoidotomy combined with endoscopy to magnify the operative field allows a far better site exposure, and the maneuverability of the endoscope is significantly increased. Endoscopy is recommended in larger tumor excision, while in cases of a tumor with limited dimensions, transoral coronoidotomy is sufficient to expose the medial masticator space. In our opinion, this approach may present some limitations: posteriorly, it does not allow control of the retrostyloid space and, cranially, it does not allow safe visualization and working beyond the caudal third of the pterygoid plates, and therefore, it does not allow access to the middle cranial fossa. Nevertheless, the lateral and medial limits of working space are easily identified: the mandibular ramus and the superior pharyngeal constrictor are perfectly visible; also, the terminal branches of the mandibular nerve and the internal pterygoid muscle can be easily identified. All considered, this surgical technique seems to allow a safe and predictable surgical access to venous malformations and benign tumors of the medial masticatory space with limited invasiveness.

In the context of malignant tumors, the applicability of this technique is limited to early-stage T1 or T2, particularly to those that do not extend into the retrostyloid space or cranially beyond the caudal third of the pterygoid plates. The main reason for such limitation is the insufficient space for visibility and surgical maneuverability, even with the use of magnifying systems. Because of these limitations, it is not possible to access the middle cranial fossa, thus ensuring procedural safety, margin control, and the required surgical radicality. In the case of advanced stage T3 or T4, with infiltration of the superficial and deep layers, and possible positive lymph node localization; however, the technique is contraindicated because its execution would pose the risk of manipulating tissues and structures that need to be eliminated during the demolition phase, with subsequent difficulty and compromise of the reconstructive phase. In such cases, a methodological approach that is different from the oncological and surgical management of advanced cases is preferred.

## Conclusion

L-shaped mandibular coronoidotomy, with or without endoscopic magnification, is a minimally invasive approach, highly suitable for the removal of a well-defined lesion of the medial masticator space. Intralesional injection of n-butyl-2-cyanoacrylate in a venous malformation avoids the risk of bleeding and allows the surgeon to complete the surgical procedure in a predictable and safe way. The described surgical technique is easily reproducible; it offers the opportunity to thoroughly explore the medial masticator space with limited surgical risks for the patient and noticeable benefits for the surgeon.

## Data Availability

The original contributions presented in the study are included in the article/Supplementary Material, further inquiries can be directed to the corresponding author.
