# Supplementary figures and images for: Minimally invasive transoral surgical approach to the medial compartment of the masticator space for venous malformation and benign tumor removal
Source: Front Surg. 2026 Apr 7;13:1691677. doi: 10.3389/fsurg.2026.1691677 (PMC13095585; doi:10.3389/fsurg.2026.1691677)

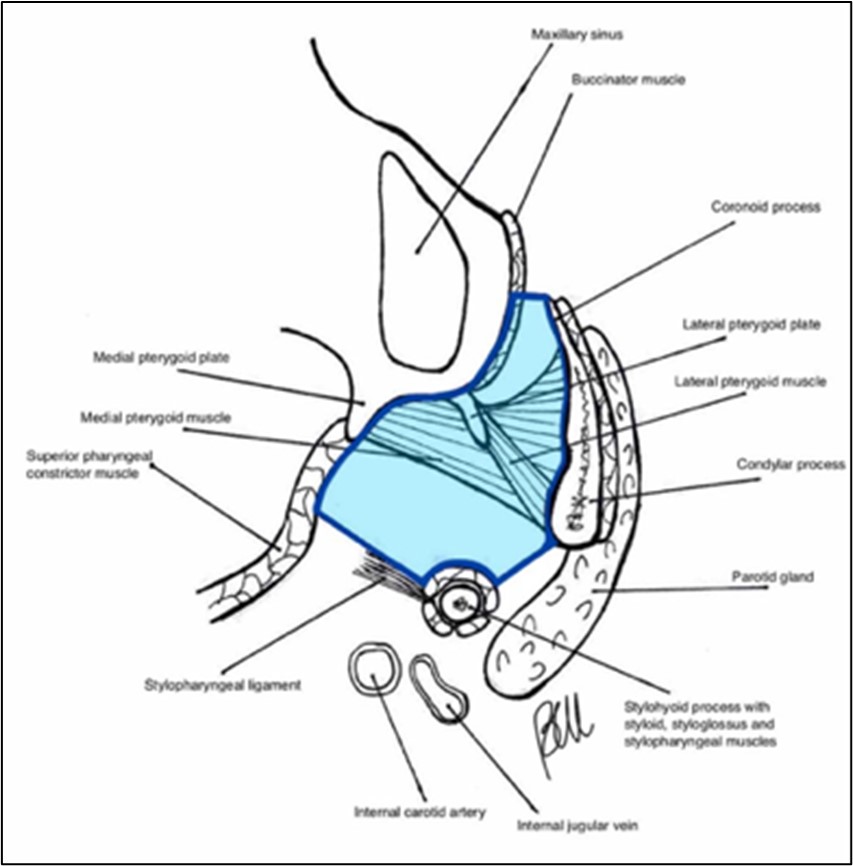

Supplement: Supplementary file 1 [file Image1.jpeg]

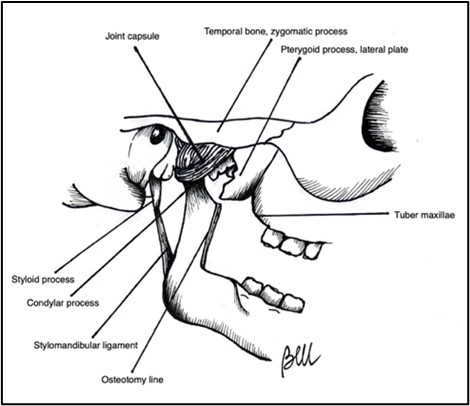

Supplement: Supplementary file 2 [file Image2.jpeg]

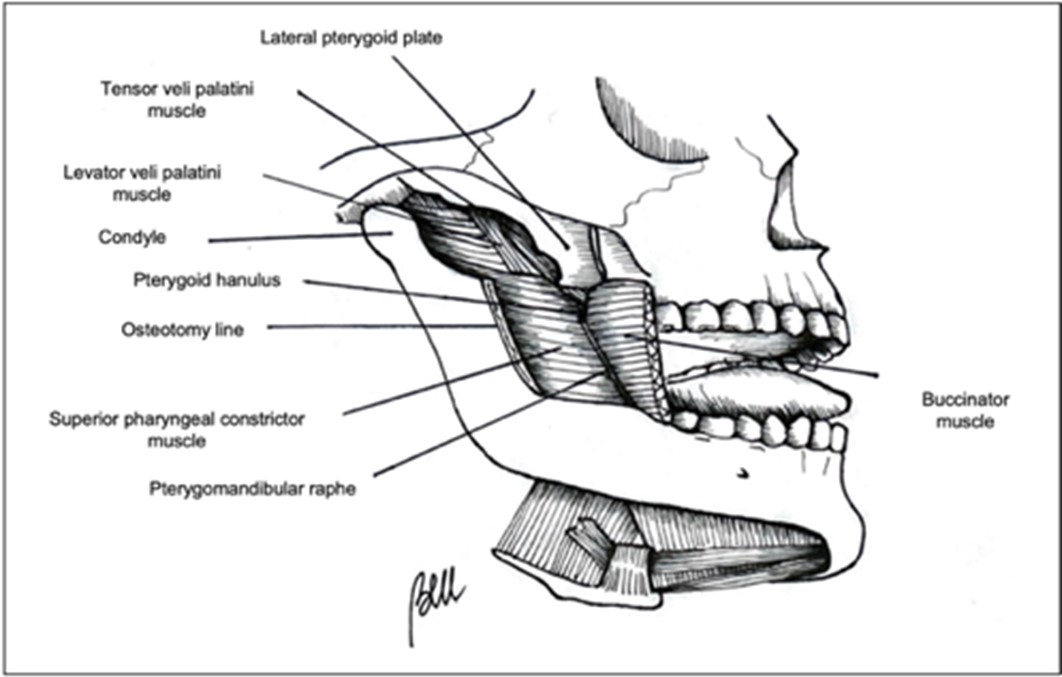

Supplement: Supplementary file 4 [file Image4.jpeg]
